# Critical Review of Gaps in the Diagnosis and Management of Drug-Induced Liver Injury Associated with Severe Cutaneous Adverse Reactions

**DOI:** 10.3390/jcm10225317

**Published:** 2021-11-15

**Authors:** Marina Villanueva-Paz, Hao Niu, Antonio Segovia-Zafra, Inmaculada Medina-Caliz, Judith Sanabria-Cabrera, M. Isabel Lucena, Raúl J. Andrade, Ismael Alvarez-Alvarez

**Affiliations:** 1 Unidad de Gestión Clínica de Gastroenterología, Servicio de Farmacología Clínica, Instituto de Investigación Biomédica de Málaga-IBIMA, Hospital Universitario Virgen de la Victoria, Universidad de Málaga, 29071 Malaga, Spain; mvilpaz@uma.es (M.V.-P.); haoniu@uma.es (H.N.); anseza9@gmail.com (A.S.-Z.); imcaliz@uma.es (I.M.-C.); sanabriajudith@hotmail.com (J.S.-C.); andrade@uma.es (R.J.A.); iaalvarez@uma.es (I.A.-A.); 2 Centro de Investigación Biomédica en Red en el Área Temática de Enfermedades Hepáticas y Digestivas (CIBERehd), 28029 Madrid, Spain; 3 Plataforma ISCIII de Ensayos Clinicos, UICEC-IBIMA, 29071 Malaga, Spain

**Keywords:** drug-induced liver injury, severe cutaneous adverse reactions, hypersensitivity, gaps, causality assessment, diagnosis, management, clinical trial, immune response

## Abstract

Drug-induced liver injury (DILI) encompasses the unexpected damage that drugs can cause to the liver. DILI may develop in the context of an immunoallergic syndrome with cutaneous manifestations, which are sometimes severe (SCARs). Nevirapine, allopurinol, anti-epileptics, sulfonamides, and antibiotics are the most frequent culprit drugs for DILI associated with SCARs. Interestingly, alleles HLA-B*58:01 and HLA-A*31:01 are associated with both adverse reactions. However, there is no consensus about the criteria used for the characterization of liver injury in this context, and the different thresholds for DILI definition make it difficult to gain insight into this complex disorder. Moreover, current limitations when evaluating causality in patients with DILI associated with SCARs are related to the plethora of causality assessment methods and the lack of consensual complementary tools. Finally, the management of this condition encompasses the treatment of liver and skin injury. Although the use of immunomodulant agents is accepted for SCARs, their role in treating liver injury remains controversial. Further randomized clinical trials are needed to test their efficacy and safety to address this complex entity. Therefore, this review aims to identify the current gaps in the definition, diagnosis, prognosis, and management of DILI associated with SCARs, proposing different strategies to fill in these gaps.

## 1. Introduction

Idiosyncratic drug-induced liver injury (DILI) is an unpredictable, host-dependent, generally not dose-related and not reproducible in animal models adverse reaction that drugs, herbal, and dietary supplements can cause to the liver [1].

DILI manifests in a variety of clinical presentations, including hypersensitivity features, which are present in a small proportion of patients. Indeed, in different DILI registries in which usually liver damage is the predominant adverse reaction, the prevalence of these hypersensitivity manifestations varies from 14% to 25% [2,3]. On the other hand, DILI can occur in the framework of an immunoallergic syndrome, in which skin reactions are the most significant manifestations. Indeed, severe cutaneous adverse reactions (SCARs) can manifest in a wide range of heterogeneous clinical presentations, including Stevens–Johnson syndrome (SJS), toxic epidermal necrosis (TEN), and more commonly, drug reaction with eosinophilia and systemic symptoms (DRESS), in which the liver is the organ most frequently affected. Characterization of liver damage in the setting of SCARs is a subject yet undefined, since there is no agreement about the criteria used for the definition of liver injury in the context of immunoallergic reactions. Moreover, the diverse criteria for DILI definition adopted across different publications hinder the comparisons between studies and make it very difficult to gain further insight on these complex hypersensitive cutaneous adverse reactions [4]. Another important challenge for clinicians is the lack of specific biomarkers in DILI, which makes the diagnosis reliant on the careful exclusion of competing etiologies of liver damage. Therefore, as there is no gold standard, the approach to causality assessment usually requires a panel of experts, which is a trustworthy method but with inherent weaknesses [5].

Moreover, in DILI cases associated with cutaneous adverse drug reactions (ADRs), liver involvement is not usually accurately assessed when skin injuries are predominant and represent the first clinical manifestation, which is partially due to the lack of information regarding liver injury in the SCARs cohorts retrospectively analyzed [6,7,8,9,10,11,12,13,14].

Although there are significant differences in which drugs are most likely to cause SCARs versus DILI [15], a number of different causative drugs have been identified as responsible for SCARs associated with DILI (Table 1). The most frequent culprit drugs might vary to some extent across countries, which may reflect differences in prescription habits and host susceptibility. These differences are most striking for SJS/TEN. In the largest series of liver injury and SJS/TEN to date involving 36 patients from a DILI registry in India, phenytoin and nevirapine (not commercialized in Europe) were the most frequently responsible drugs [16]. However, retrospective studies from Korea and Singapore identified allopurinol as the most frequent culprit drug for SJS/TEN associated with DILI [7,17]. Nevertheless, for DRESS syndrome, the responsible drugs and drug classes were the same in most studies: allopurinol, anti-infectives, aromatic anticonvulsants, and nonsteroidal anti-inflammatory drugs (NSAIDs) [6,7,8,9,10,12,13,14,17,18,19]. In the multinational Registry of Severe Cutaneous Adverse Reactions (RegiSCAR), which is the largest international SCARs registry, the most frequent culprit drugs implicated in DRESS were anticonvulsants (carbamazepine, phenytoin, lamotrigine), allopurinol, sulfonamides and antibiotics [20]. In this prospective study, liver involvement was found in 75% of cases.

The immune checkpoints inhibitors (ICI) by targeting immune checkpoints favor a vigorous and sustained immune response and represent a new paradigm in the treatment of advanced cancer. The ICI block the CTLA-4 and PD-1 pathways [21]. However, these drugs have also been associated with frequent immune-related adverse events (irAEs) in several organs of the body, including skin, lung, endocrine systems, the gut, and the liver [21,22]. A few studies reported DRESS syndrome (with liver involvement) as an adverse reaction of ipilimumab, which is an anti-CTLA-4 antibody in metastatic melanoma [23,24]. A recent study characterizing the cutaneous irAEs submitted to the U.S. Food and Drug Administration Adverse Event Reporting System (FAERS) showed that 7.5% of the SCARs reported for anticancer drugs were due to ICI. Noteworthy, 11% of ICI-induced SCARs showed concomitant hepatobiliary damage [25].

Interestingly, recent studies reported that candidate drugs for the treatment of coronavirus disease 2019 (COVID-19), such as azithromycin, hydroxychloroquine, ivermectin, or tocilizumab [26,27,28,29], could be DRESS culprits.

Immediate withdrawal of the causative drug is the first and critical step in the management of DILI. Under the rationale that SCARs are immunoallergic adverse reactions to drugs, steroids remain the cornerstone of therapeutic approach of these patients. Furthermore, clinicians are prone to use corticosteroids in severe DILI or DILI associated with prominent hypersensitivity features, including autoimmune-like hepatitis [3,30]. However, the beneficial effect of steroids in liver injury has neither been assessed in the setting of SCARs nor in DILI patients within properly designed controlled clinical trials [31,32].

Therefore, this review aims to identify and discuss the current research gaps in the diagnosis and management of DILI associated with SCARs, focusing on the disparities of the definition criteria used in the studies conducted to date. Finally, different approaches will be put forward to fill in those gaps.

## 2. How Should We Define DILI in the Setting of SCARs?

Liver is the internal organ most frequently involved in SCARs [34]. However, and despite the availability of national clinical guidelines for the diagnosis, management, and prevention of SCARs [35,36], there is no consensus about the definition of liver injury in the context of SCARs, which leads to a troublesome diagnosis and treatment of this complex entity and represents a major gap to be addressed.

In the past years, international groups have worked toward the correct identification of conditions involving DILI associated with SCARs, such as the DRESS syndrome. Thus, the criteria and scoring system proposed by the RegiSCAR group are the currently most accepted definition for DRESS [37]. RegiSCAR criteria defined liver injury according to those proposed by Benichou et al. [38] more than 30 years ago, which were updated in 2011 [39]. Thus, the biochemical criteria to define DILI currently accepted are alanine aminotransferase (ALT) > 2 times the upper limit of normality (ULN) on at least two successive dates, conjugated bilirubin > 2xULN on at least two successive dates, or aspartate aminotransferase (AST), total bilirubin (TBIL), and alkaline phosphatase (ALP) all > 2xULN [20]. On the other hand, a Japanese consensus group has proposed similar diagnostic criteria for DRESS, also named drug-induced hypersensitivity syndrome, including the human herpesvirus 6 reactivation as a distinctive clinical criterion to confirm the diagnosis [40]. Nonetheless, this latter criterion has not been adopted in other countries outside Japan and other Asian countries nor other international registries [41], yet its validity needs to be confirmed. The Japanese criterion for liver abnormalities was defined as ALT > 100 U/L in the so-called Japanese severe cutaneous adverse reaction (J-SCAR) criteria [40]. In 2010, the International Serious Adverse Events Consortium organized a meeting of experts in drug-induced skin injury to define the minimum phenotypic criteria for the selection and recruitment of patients with SCARs. Despite the availability of the above-mentioned diagnostic criteria, the expert working group expressed their concerns in relation to these definitions, especially with regard to liver injury definition in the context of SCARs, as it might merely reflect an abnormal elevation in liver enzymes but not real hepatitis [42].

This low specificity in the definition of liver injury previously commented contrasts with the updated DILI criteria proposed by a multidisciplinary expert group in the framework of the International Serious Adverse Events Consortium in 2011 [39]. This panel agreed to raise the thresholds and proposed the most widely accepted and standardized definition for DILI (ALT ≥ 5xULN, or ALP ≥ 2xULN, or ALT ≥ 3xULN and TBIL > 2xULN). However, most of the studies performed after this definition was available continued using the RegiSCAR criteria to define liver injury in the context of SCARs [7,8,18], while only few studies have adopted the more specific definition of liver injury [6]. In light of the evidence, the adoption of standardized criteria to define liver injury in the context of SCARs remain as an urgent need.

The adoption of the stringent DILI diagnostic criteria proposed by the aforesaid expert consensus [39] would represent a step toward this standardization. One of the strengths relies on their specificity by discarding mild liver injuries related to alternative causes. These are the standardized criteria set forward in international clinical practice guidelines and position papers on DILI and are currently used in the international prospective DILI registries [2,3,32,43,44,45].

On the other hand, the presence of hypersensitivity features might not be considered a specific criterion in the diagnosis of idiosyncratic DILI. The prevalence of hypersensitivity features such as eosinophilia among DILI patients ranged from 11 to 23% across prospective DILI registries [2,3,45]. In a study from the Spanish DILI Registry, the presence of eosinophilia was related to a better prognosis [46]. Furthermore, another investigation yielded the lack of association of eosinophil infiltration and age or sex regarding histological findings, diminishing its role in the diagnosis [47]. Indeed, along the causality assessment scales to help with the diagnosis, the Digestive Disease Week-Japan causality scale includes a drug lymphocyte stimulation test among its items [48], while the Maria-Victorino scale refers to the presence of immunoallergic signs [49].

## 3. A Dual Causality Assessment for SCARs and DILI: Where Do We Stand?

Due to the lack of biomarkers or specific tests in the diagnosis of DILI associated with SCARs, the careful exclusion of other competing liver causes becomes crucial to identify a specific drug as the cause of the adverse reaction. Thus, the usefulness of causality assessment tools to determine the role of a suspected drug is particularly relevant, although this process is challenging in those cases when multiple suspected drugs are involved.

Currently, the relationship between drugs and related adverse events can be evaluated by different methodologies: expert judgment, probabilistic methods, algorithms, or scales. The consensus judgment provided by experts based on clinical experience and thorough analysis of competing etiologies tends to maximize the specificity and is currently considered as the best standard [39,50]. However, the expert consensus presents limitations, such as the poor inter-rater reliability, the reliance on subjective opinions based on physician’s skills and awareness, and the absence of an expert panel in daily practice, which limits its generalizability [51,52].

In addition to the expert’s consensus agreement, there are complementary causality assessment tools available to help in the identification of the causative agent responsible for the DILI episode associated with SCARs. Nonetheless, despite this availability, these tools have shortcomings in the correct evaluation of DILI associated with SCARs (Table 2). One of the tools that has been used to establish the causal relationship in the past decades is the Naranjo scale [53]. Although this tool was designed for the assessment of adverse drugs reactions, it presents several drawbacks. First, it has several items subjected to subjective interpretations, which diminishes its reproducibility. Furthermore, it is a not organ-specific scale; thus, the type of liver injury or presence of hypersensitivity features are not defined, and it contains questions not relevant to idiosyncratic reactions [54]. Altogether, this scale is clearly limited to be used in the context of hepatotoxicity episodes. Another available scale is the one developed by the World Health Organization and the Uppsala Monitoring Centre (WHO-UMC). This method stresses the importance of the clinical–pharmacological aspects of the case history and the quality of the documentation of the observation. Conversely, based on the premise that this tool is used to detect unexpected adverse reactions, previous knowledge and statistical chance play a less important role [55]. Adverse reactions are judged on the basis of certain assessment criteria (temporal relationship, abnormal laboratory tests, dechallenge and rechallenge, and plausibility and absence of other drugs or diseases). Its main advantage is that it can be easily used and customized at a local level. However, this tool presents with a very low sensitivity when compared to other scales [56], which is a major disadvantage that compromises its utility in the causality assessment of DILI in the context of SCARs.

More recently, a group of experts built up an algorithm of drug causality in epidermal necrolysis, the so-called ALDEN, to evaluate the causality of medications in SJS or TEN [57]. However, this specific algorithm is focused on these entities and does not take into account the liver damage in the systemic adverse reaction [16], thus limiting its usefulness in the assessment of DILI associated with SCARs.

On the other hand, the RegiSCAR study group has developed its own scoring criteria to conduct a case assessment [37]. They scored positively the presence of hypersensitivity features (eosinophilia, lymphopenia) and both the skin and internal organ involvement. Liver damage was subsequently detailed as a two-fold elevation above the ULN of either ALT, conjugated bilirubin, or AST, total bilirubin, and ALP all together [59]. However, the use of these less stringent thresholds below the current criteria established [39] may limit the specificity of this scoring system in the identification of a DILI episode in a patient with SCARs.

The most extended causality assessment tool for DILI is the validated Council for International Organizations of Medical Sciences/Roussel Uclaf Causality Assessment Method (CIOMS/RUCAM) scale [58]. This scale is composed of seven key features (time to onset, risk factors, course of the reaction, concomitant drugs, exclusion of non-drug causes, previous information about hepatotoxic potential, and response to rechallenge) and has shown a high sensitivity, specificity, and predictive values, although the validation process was questioned due to the lack of a gold standard [58]. Nonetheless, it shows some limitations when evaluating causality in patients with DILI associated with SCARs. Neither immunoallergic features (such as skin rash, fever, eosinophilia) nor genetic variations associated with DILI and hypersensitivity reactions predisposition are incorporated in the scoring system [60]. Consequently, the lack of these specific items highlights the need for further refinement to establish a more accurate causal relationship in DILI cases with SCARs. In 1997, Maria and Victorino developed a simplified causality score that scored positively on the presence of immunoallergic features [49]. The rationale for including this criterion was the high specificity to detect DILI, although no further criterion related to systemic reactions was added.

The Digestive Disease Week-Japan (DDW-J) scale was developed by the Japan Society of Hepatology [48]. Despite it being based on the CIOMS/RUCAM scale, some items were modified, i.e., the temporal relationship is scored differently when compared to the CIOMS/RUCAM scale, and the concomitant drugs item was omitted. Notably, this scale included two new items related to extrahepatic hypersensitivity manifestations. Thus, a positive drug lymphocyte stimulation tests and the presence of eosinophilia were positively scored [48]. Notably, the scale was reevaluated to check its performance in recent DILI cases. Although the authors acknowledged that the scale needs to be updated to reflect liver injury caused by novel drugs such as immune checkpoint inhibitors, the scale remains as a tool with a fair diagnostic performance [61]. However, its limited access in terms of difficulties of reading it online and the non-usual performance of drug lymphocyte stimulation tests in the clinical practice have prevented its generalizability outside Japan [60,61].

A different approach to delve into the impact of both individual and drug-specific factors on the pathogenesis of DILI associated with SCARs is the use of *in vitro* systems such as patient-derived cells. Monocyte-derived hepatocyte-like (MH) cells from DILI patients have been proposed for the identification of drug-specific immune-mediated reactions [62], although this system requires external validation. Traditionally, the lymphocyte transformation test (LTT) has been useful to diagnose drug hypersensitivity *in vitro*, despite it exhibiting limited sensitivity and specificity depending on the reaction and the culprit drug [63,64]. A few years ago, a modified LTT measuring granzyme B and cytokines (interleukin (IL)-2, IL-5, IL-13, and interferon (IFN)-γ) production was proposed to diagnose DILI associated with an adaptive immune response and identify the responsible drug by using peripheral blood mononuclear cells (PBMC) from subjects enrolled in the U.S. Drug-Induced Liver Injury Network (DILIN). However, this modified version of LTT could not reliably establish causality in most of the patients, except in one particular case exposed to isoniazid [65]. The use of patient-derived keratinocytes would also be an interesting approach to identify risk factors associated with liver injury related to immune-mediated cutaneous reactions [66]. Recent data suggest that DILI-associated genes are involved in the onset and progression of drug-induced skin toxicity and keratinocytes and hepatocytes may share similar responsiveness to hepatotoxic drugs [67].

Therefore, the gap to address in the causality assessment of DILI associated with SCARs relies on the need to standardize the use of an easy-to-use scale in the clinical setting that evaluates both hepatic and extrahepatic manifestations. Novel proposals, such as the modified electronic version of the CIOMS/RUCAM scale, namely Revised Electronic (version) of Causality Assessment Method (RECAM) [68] (Supplemental Table S1), which scores positively the presence of SCARs, are appealing. The incorporation of hepatic damage parameters to conduct a holistic evaluation could be considered in a revised version of the ALDEN.

## 4. The Role of HLA Genes in Adverse Drug Reactions: Valid Genetic Markers?

**Figure 1:**
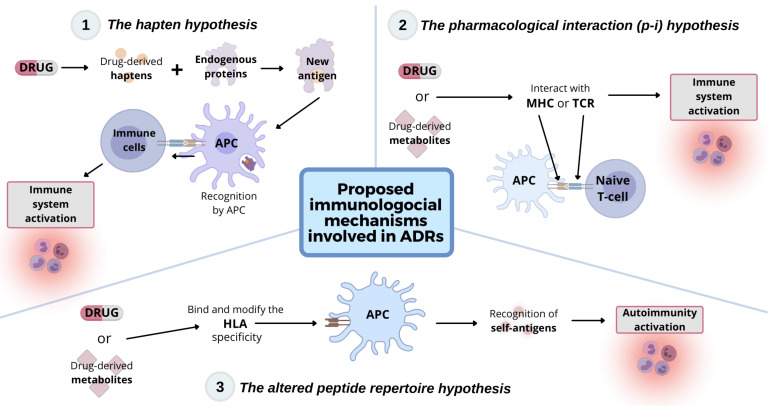
Immune system association in DILI with SCARs: The three main hypotheses regarding the immunological mechanisms involved in ADRs are: (1) The hapten hypothesis, (2) The pharmacological interaction (p-i) hypothesis, and (3) The altered peptide repertoire hypothesis.

The occasional occurrence of DILI with SCARs strengthens the immunological mechanisms underpinning these manifestations and implications for therapy [69]. Increasing evidence points to a crucial role for the innate and adaptive immune system in the pathogenesis of liver and cutaneous adverse reactions [70]. Different hypotheses for the involvement of the immune response in the development of these ADRs have been proposed (Figure 1): (I) the hapten hypothesis, in which drug-derived metabolites form hapten peptides with endogenous proteins that activate the immune system [71]; (II) the pharmacological interaction (p-i) hypothesis, in which specific drugs interact non-covalently with major histocompatibility complex (MHC) molecules, activating the immune system [72], and (III) the altered peptide repertoire hypothesis, in which specific drugs lead to the attachment of endogenous peptides to the wrong human leucocyte antigen (HLA), provoking autoimmunity [73]. Inflammation has also a very important role in the development of liver damage, as well as concomitant inflammatory disorders, which can change the cytokine environment in favor of an immune response, promoting organ injury [74].

There is also growing evidence that DILI and different ADRs are dependent on the superposition of many different factors such as gender, age, genetics, and environmental and physiological factors [75,76,77]. Therefore, unless different factors concurred simultaneously, ADRs will not develop.

**Figure 2:**
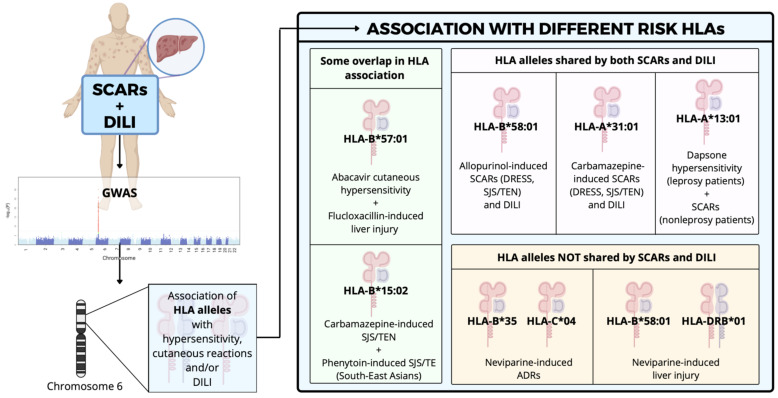
Different GWAS-determined HLA alleles linked to the risk of developing ADRs. There are three main types of associations: Some overlap in HLA associations, where the same allele is linked to the risk of developing ADR due to different drugs (green boxes); HLA alleles that are shared by both SCARs and DILI and trigger both ADRs (pink boxes); and HLA alleles not shared by SCARs and DILI, where different ADRs due to the same drugs are linked to different HLA alleles (orange boxes). DILI, Drug-Induced Liver Injury; SCAR, Severe Adverse Cutaneous Reaction; ADR, Adverse Drug Reaction; GWAS, Genome-Wide Association Studies; HLA, Human Leukocyte Antigen; DRESS, Drug Reaction with Eosinophilia and Systemic Symptoms; SJS, Stevens–Johnson Syndrome; TEN, Toxic Epidermal Necrosis.

This might partially explain why the disease is so infrequent despite several genome-wide association studies (GWAS) reporting associations between HLA alleles and the predisposition to ADRs related to a number of drugs [78]. The discovery of these associations enlightened the possibility of predicting ADRs through pharmacogenetic screenings [79]. Examples of some of these associations can be observed in Figure 2.

Some investigations have suggested an overlap of HLA alleles of risk, where one allele may increase the predisposition to different drug-induced hypersensitivity reactions and phenotypes [80]. For example, carriage of the HLA-B*57:01 allele increases by 80-fold the risk of flucloxacillin-induced liver injury and at the same time is strongly associated with abacavir hypersensitivity [81,82]. Currently, HLA-B*57:01 genotyping prior to abacavir prescription is mandatory [83,84], which has been stated as a cost-effective intervention [85]. In the same way, carriage of the HLA-B*15:02 allele has been associated with an approximately 100-fold risk of developing SJS/TEN with carbamazepine [86] and with a 28-fold risk of developing SJS/TEN with oxcarbazepine in Chinese and Thai populations [87].

At the same time, the HLA-B*15:02 allele is associated with phenytoin-induced SCARs [88]. In fact, this allele is recommended to be tested in patients of South East Asian descent to prevent a carbamazepine-induced SJS/TEN [89], since the clinical utility of pre-prescription HLA-B*15:02 screening has been recently demonstrated by different studies in Taiwan [90,91].

Regarding drug hypersensitivity with cutaneous manifestations, there can be HLA alleles shared by both SCARS and DILI. Carriers of the HLA-B*58:01 allele showed increased susceptibility to allopurinol-induced SCARs, both DRESS and SJS/TEN [92,93,94]. Interestingly, in a recent 10-year multi-center prospective study about DILI associated with SCARs in Taiwan, a higher positive rate of HLA-B*58:01 in patients with allopurinol-induced DILI with SCARs than in those with DILI without SCAR has been observed (92.3% vs. 16.7%, *p* < 0.001) [33].

Moreover, it has been reported that HLA-A*31:01 was the strongest predictor of carbamazepine-induced SCARs, as well as the predisposition to carbamazepine-induced liver injury, indicating that HLA-A*31:01 carriage is a shared risk factor for SCARs and DILI induced by this drug [95]. In the same way, the HLA-A*13:01 allele has been associated with the development of the dapsone hypersensitivity syndrome (principally characterized by skin lesions and elevated transaminases) in leprosy patients [96] and with dapsone-induced SCARs in non-leprosy patients [97].

However, to date, only the alleles mentioned above have been associated with both DILI and SCARs predisposition. It is unknown whether these alleles play a role in the severity of liver injury manifested in cases of DILI associated with SCARs or whether they constitute a prognostic factor for DILI in patients with SCARs. Further GWAS studies are needed to establish genetic risk factors associated with both SCARs and DILI hypersensitivity reactions induced by other drugs.

Alternatively, some drugs are well known to cause both DILI and SCARs, but the genetic risk factors for cutaneous reactions appear to be different from those related to liver injury. Nevirapine, a widely prescribed antiretroviral treatment, has been associated with both DILI and cutaneous ADRs. Carriage of the HLA-B*35 and HLA-C*04 alleles increase by 2.45-fold and 2.63-fold, respectively, the risk of nevirapine-induced cutaneous ADRs. On the other hand, carriage of the HLA-B*58:01 and HLA-DRB*01 alleles increase by 3.51-fold and 2.94-fold, respectively, the risk of nevirapine-induced liver injury [98]. However, interestingly, a population-based study from Western Australia found an association between the HLA-DRB1*0101 allele and nevirapine-induced rash-associated hepatitis in those patients with a CD4 ≥ 25% [99].

## 5. Prognostic Factors in DILI and SCARs: From the Model to the Clinical Setting

The correct identification of prognostic factors is paramount in order to provide an adequate medical approach to the management of these reactions.

One of the well-established models to predict death is SCORTEN, which has been tested using data from patients enrolled in the RegiSCAR. This validated score includes suspected prognostic factors for hospital mortality (age ≥ 40 years, heart rate ≥ 120 per minute, cancer or hemopathy, body surface area involved > 10%, serum urea level > 10 mmol/L, serum bicarbonate level < 20 mmol/L, and serum glucose level > 14 mmol/L) [100] and showed an acceptable performance in SJS/TEN patients [101]. Interestingly, the accuracy of this score has been recently evaluated through a systematic review and meta-analysis, concluding that the predicted mortality did not differ from the observed, and therefore, it could be deemed as a reliable estimator for the fatal outcome [102]. In the RegiSCAR study, investigators presented a simplified model (the Auxiliary score) accounting for age (categorized in age groups: ≤30, 31 to 55, 56 to 75, and ≥75 years; >30% of skin detachment and presence of cancer). This model yielded a similar predictive performance to the SCORTEN score but had extended applicability due to its simplicity. In addition, the authors noted that there was some room for improvement in the SCORTEN model by the inclusion of a comorbidity score [101].

Nonetheless, one of the limitations of these scores is their restriction to SJS/TEN entities. More importantly, liver involvement is not considered among the prognostic risk factors analyzed. In a systematic review including both observational studies and case reports, Lorenz et al. (2012) found that in patients treated with dapsone who developed hypersensitivity reactions, hepatitis was significantly associated with a fatal outcome [103]. Further, in an ambispective study with 36 SJS/TEN patients, Devarbhavi et al. (2016) identified the presence of ascites, elevated bilirubin, and a higher Model for End-Stage Liver Disease score as significant predictors of mortality [16].

The presence of liver involvement and associated comorbidities is a critical factor that determines outcome in SCARs, particularly in DRESS and SJS/TEN [104]. This central role of liver involvement as a prognostic factor indicates the need for further refinement in predictive models to account not only for parameters related to the skin injury but awareness of the importance of internal organ involvement, specifically liver damage. The translation of a refined prognostic model to the clinical setting will represent a step toward an enhanced decision-making process and personalized medicine in the management of patients with DILI associated with SCARs.

## 6. Management of DILI Associated with SCARs: In the Quest of High-Quality Evidence

When a patient is diagnosed with DILI associated with SCARs, the main steps to be taken in the management of the disease are the identification and immediate withdrawal of the offending drug, which is followed by the needed administration of supporting treatment. Nonetheless, regarding symptomatic treatment, clinical trials designed to test the efficacy of specific treatments are still scarce, consequently leading to a gap of high-quality evidence in the treatment of this disorder.

Treatment with systemic corticosteroids is currently considered the most successful treatment, which is based on the role played by T cells in the pathogenesis of drug-induced hypersensitivity syndrome [105,106]. Nonetheless, the use of steroids relies on the findings from observational studies. In a Korean study including 136 patients with drug-induced systemic hypersensitivity reactions, systemic corticosteroids administration did not lead to improvements in liver dysfunction recovery, duration of rash, or mortality [7]. In a systematic review of 20 allopurinol-induced DRESS case reports, treatment mostly consisted in the administration of systemic corticosteroids, but a great variability in the route of administration, dose, or treatment duration was evidenced [107]. Another retrospective study compared the treatment with topical and systemic corticosteroids in 38 DRESS patients from the RegiSCAR. The clinical course in both groups was similar, but patients treated with systemic corticosteroids suffered complications more frequently (infections, septicemia) and had more relapses and prolonged hospitalization, which led to the conclusion that systemic corticosteroids treatment should be reserved only for severe DRESS cases [108]. In the Spanish Guidelines for the diagnosis, management, and treatment of DRESS, recommendations are the use of topical corticosteroids for SCARs and continual monitoring and close follow up in mild DILI cases, while in serious DRESS cases with a more severe internal organ involvement, the recommendation is to administer systemic corticosteroids [35]. However, this latter recommendation is based on two retrospective cohort studies that found benefits in corticosteroids administration in severe DILI patients with hyperbilirubinemia [109,110].

In addition to the widely established treatment with corticosteroids, especially for SCARs, the use of intravenous immunoglobulins (IVIg) as an immunomodulatory agent deserves further attention. A group of experts of the French Society of Dermatology developed a decisional tree where the use of IVIg was restricted to severe DRESS cases with life-threatening signs or a major viral reactivation [111]. The debate about the risk–benefit ratio of using IVIg in DRESS patients is based on data from a prospective study with a very limited sample of patients. Thus, in a French study including six patients with severe DRESS, in only one patient was the disease remitted, while the other five patients were dropped out due to severe adverse reactions [112]. In SJS and TEN patients, the efficacy of IVIg still remains controversial. In a retrospective study, the combined treatment with corticosteroids and IVIg showed benefits over only corticosteroids treatment in SJS and TEN patients by reducing the time to arrest progression and hospitalization time, although no differences in mortality rate were seen [113].

In a systematic review including mostly observational studies, the authors concluded that treatment with IVIg did not reduce mortality risk when compared to supportive care in patients with SJS and TEN, neither when data were meta-analyzed at a study nor patient level [114]. However, a more recent network meta-analysis combining observational studies and clinical trials yielded that when compared to supportive care, the combination therapy with corticosteroids and IVIg significantly lowered SCORTEN-based standardized mortality risk in these patients [115].

In those patients with contraindications for steroids treatment, cyclosporine administration has been considered as a candidate therapeutic option, despite its known nephrotoxicity [116]. The evidence for using cyclosporine mainly relies on case series reports [117,118] and retrospective studies. One study reported the therapeutic benefits in terms of survival of cyclosporine over IVIg in 35 patients with SJS/TEN [119]. In the same line, cyclosporine treatment in a limited sample of DRESS patients was associated with shorter time to cessation of progression and duration of hospital stay when compared to patients treated with corticosteroids [120].

Evidence from randomized clinical trials is still scarce. Findings from one open single-arm trial with 29 patients with SJS/TEN receiving cyclosporine suggested a beneficial effect of treatment on decreasing mortality [121]. More recently, another open trial aimed to compare the efficacy of TNF-α antagonists (etanercept) versus corticosteroids in a sample of 96 patients with SJS/TEN. Patients who received etanercept showed a shorter time for complete skin healing but no differences in mortality rate [122]. However, up to date, no high-quality randomized clinical trials have been conducted in the context of DILI associated with SCARs with a standardized methodology. A recent systematic review and meta-analysis concluded that randomized clinical trials in the context of DILI lack standardized methodologies and endpoints and highlighted the need for better performed randomized clinical trials (RCTs) [123].

Hence, in the light of the scientific evidence, and bearing in mind that the treatment of this complex and heterogeneous disorder comprises the treatment of both liver and skin injuries, it is necessary to conduct high-quality evidence-based studies to reach conclusions about the efficacy and safety of the above-mentioned treatments and other novel systemic immunomodulating treatments, such as tumor necrosis factor inhibitors.

## 7. Future Perspectives and Challenges

**Figure 3:**
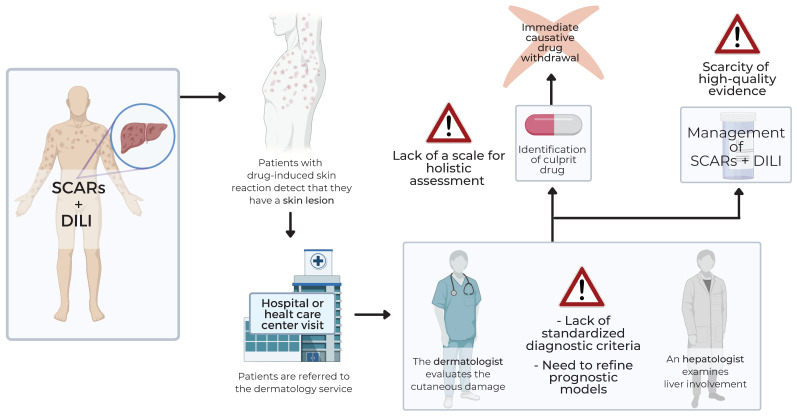
Identified gaps in the pathway of diagnosis and management of DILI associated with SCARS. DILI, Drug-Induced Liver Injury; SCAR, Severe Adverse Cutaneous Reaction.

This study was conceived as an opportunity to critically revise the current gaps in the scientific literature in a rare but potentially fatal condition, as DILI is associated with SCARs (Figure 3). Despite the extensive research achievements, which should be duly acknowledged, the current diagnosis of DILI associated with SCARs is limited to clinical case definition and the use of causality assessment tools. In addition, there is a lack of well-performed clinical trials to test the efficacy and safety of the current and novel therapeutic options. Therefore, we consider there is room for improvement in these areas to fill the gaps described in this manuscript.

First, diagnostic criteria are heterogeneous. Most studies in the context of DILI associated with SCARs use the RegiSCAR or the J-SCAR criteria. However, these criteria lack specificity, as thresholds proposed may reflect an abnormal liver function but not a real DILI. To overcome this limitation, in a DILI expert meeting held in 2011, the panel aimed to harmonize the criteria and standardize a definition. The consensus reached led to a rise of the previous thresholds [39]. However, these criteria were not adopted by international registries such as the RegiSCAR to define entities such as DRESS, in which liver involvement is not only frequent but can determine to some extent the severity of the condition. Therefore, one proposal to fill this gap is to plan a multidisciplinary and international expert meeting including different profiles involved in the diagnosis and management of the condition (hepatologists, dermatologists, clinical pharmacologists, immunologists). This panel should agree on the harmonized criteria and the tests to be performed, which will allow the correct diagnosis of both liver damage and skin injury.

In addition, there is a need for new diagnostic tools that take into account the involvement of host factors in ADRs susceptibility. Numerous associations between HLA alleles and DILI/SCARs risk have been discovered in the last years. Although the majority of them have a very high negative predictive value, due to the low incidence of these ADRs, HLA testing should become a complementary test to facilitate diagnosis and prevent severe DILI/SCARs episodes [124].

In the next steps, external validation of new *in vitro* diagnostic test systems, such as MetaHeps^TM^, which uses MH cells from the affected patients, could help to identify the drug responsible for DILI/SCARs episodes in cases of polypharmacy. In this line, within the IMI2—TransBioLine project (https://transbioline.com, accessed 15 November 2021), the Work Package 2 aims to discover, validate, and qualify novel biomarkers in liver injury, which represents a step toward the future availability of diagnostic tests in the clinical setting. Moreover, some authors have suggested that the inclusion of *in vivo* diagnostics, such as the patch or delayed intradermal testing, may improve the clinical identification of immune-mediated adverse reactions and act as potential preventive biomarkers, although they are not widely available [125,126]. Obtaining a better clinical picture of the liver damage associated with SCARs would contribute to better characterize DILI in the context of SCARs and its role in the severity and outcome of the skin reaction.

With regard to causality assessment, experts’ judgment based on their experience and the exclusion of other competing etiologies remains as the best standard available up to date. The availability of a wide range of tools may help in the ascertainment of the role of a suspected culprit drug in the development of the condition, but the great variability across scales may, paradoxically, result in a disadvantage. The widely accepted CIOMS/RUCAM scale is considered the most appropriate tool to ascertain causality in DILI, but its usefulness is limited in the context of SCARs because hypersensitivity features are not scored. Therefore, this scale needs refinement to be useful in the evaluation of broad conditions. Furthermore, the time from first drug intake to the onset of the symptoms may vary according to the drug and the condition. Thus, DRESS patients exposed to antimicrobials showed a shorter latency compared to those who had taken anticonvulsants or allopurinol [127]. Likewise, latency in DRESS cases was significantly longer when compared to SJS/TEN patients [128]. These inter-patient differences should be considered when building up a new scale. In this line, the newly developed revised electronic version of the CIOMS/RUCAM scale, RECAM [68], includes one item asking for the presence or absence of DRESS or SJS. Despite this first step toward including systemic reactions, a more thoughtfully and detailed weighting of hypersensitivity features is needed to achieve a more accurate evaluation. In addition, there is a need to establish a standardized operational protocol for the use of LTT in order to allow the comparison of results between international stakeholders.

The lack of high-quality evidence-based studies in the context of DILI associated with SCARs remains as the main limitation in the evaluation of efficacy and safety of the available systemic immunomodulating treatments. Despite this, recent investigations using novel approaches, such as network meta-analysis to compare the efficacy of the different therapeutic options, have yielded that corticosteroid therapy showed benefits in treating SCARs [115], and its administration was not associated with a progression to acute liver failure in DILI patients [30]. Thus, there is an urgent and unreserved need for well-performed randomized clinical trials to test both current and novel agents that address both liver and skin injury. One critical step to fill this gap should be to rely on harmonized diagnostic criteria to ensure adequate inclusion criteria. Due to the rareness of the condition, a sufficient sample size is needed to ensure statistical power. In addition, the design of these trials would require defining precise endpoints, monitoring plans, and stopping rules. Altogether, it would allow the performance of high-quality clinical trials to reach solid conclusions about the efficacy and safety of therapeutic options.

Another complementary tool to clinical trials to retrieve information about the safety and efficacy of tested compounds would be the use of real-world evidence, in which data from different sources such as electronic health records, claims data, prescription records, and environmental data confluence and provide a holistic view of an individual’s health status [129]. Given the rarity of the condition, this evidence from outside the scope of a traditional clinical trial may provide useful information to support future clinical decisions. Moreover, as an intentional rechallenge is unacceptable from an ethical point of view, data obtained from the real-world evidence would benefit the clinical characterization of accidental re-exposures to suspected culprit agents. Therefore, data obtained from real-world evidence jointly with the experience gained in well-performed clinical trials may represent a further step toward evidence-based therapeutic decisions to manage this complex disorder.

In conclusion, there are opportunities for improvement in the diagnosis and management of DILI associated with SCARs. We have critically reviewed some of the gaps and proposed some ideas to fill them. A multidisciplinary expert consensus meeting is necessary to harmonize diagnostic criteria and causality assessment. There is a need for a consensus on a list of biomarkers and *in vitro* and *ex vivo* models to provide a confident diagnosis. The publication of specific international clinical practice guidelines would likely address most of the gaps summarized in this review.

## Figures and Tables

**Table 1:** Most common culprit drugs for DILI associated with SCARs.

Study	N (DILI+SCARs)	Top Causative Drugs	Type of Liver Injury	Mortality
**Huang et al.** [33]	158	Allopurinol (25%) Sulfamethoxazole-trimethoprim (23%) Carbamazepine (19%) Phenytoin (16%) Diclofenac (13%) Antituberculosis drugs (2.5%) Amoxicillin-clavulanate (2.5%)	Hepatocellular (37%) Mixed (33%) Cholestatic (30%)	All-cause (24%) Liver-related (6%) Skin-related (9.5%) Liver and skin-related (5%)
**Devarbhavi et al.** [16]	36	Phenytoin (22%) Nevirapine 17%) Dapsone (14%) Carbamazepine (11%) Cotrimoxazole (8%) Leflunomide (8%) Phenobarbitone (6%) Allopurinol (6%) Lamotrigine (3%) Oxcarbazepine (3%)	Hepatocellular (36%) Mixed (50%) Cholestatic (14%)	All-cause (36%)
**Lee et al.** [7]	61	Beta-lactam (26%) NSAID (16%) Allopurinol (13%) Anticonvulsants (10%) Herbal medicine (6.5%) Sulfonamide (5%) Antituberculosis (5%) Antineoplasic (5%) Quinolone (3%) Other antibiotics (3%)	-	All-cause (18%)
**Eshki et al.** [14]	9	Allopurinol (33%) Minocycline (22%) Phenobarbital sodium (11%) Methicillin sodium (11%) Phenytoin (11%) Sulfasalazine (11%)	-	All-cause (22%)
**Walsh et al.** [18]	27	Carbamazepine (22%) Phenytoin (22%) Sulfasalazine (11%) Minocycline (11%) Allopurinol (7%) Isoniazid (4%) Trimethoprim (4%) Piperacillin/tazobactam (4%)	-	All-cause (22%)
**Lin et al.** [8]	62	Allopurinol (24%) Phenytoin (16%) Dapsone (13%) Carbamazepine (12%) Beta lactam (5%) SMX-TMP (5%) Lamotrigine (3%) Sulfasalazine (3%)	Hepatocellular (19%) Mixed (27%) Cholestatic (37%) Non-defined (16%)	-
**Medina-Caáliz et al.** [19]	60	Amoxicillin-clavulanate (12%) Carbamazepine (17%) Allopurinol (8%) Lamotrigine (8%)	Hepatocellular (42%) Cholestatic-mixed (58%)	All-cause (0%) *

* Only DILI-DRESS cases.

**Table 2:** Strengths and limitations of the causality scales used in the assessment of DILI associated with SCARs.

Causality Assessment Scales	Strengths	Limitations
**Naranjo** [53]	Applicable to assess all types of ADRs Simplicity	Items prone to be interpreted subjectively Not organ-specific Not recommended for DILI assessment
**WHO-UMC** [55]	Easy-to-use Able to be customized at a local level	Low sensitivity
**ALDEN** [57]	Detailed algorithm of drug causality in epidermal necrolysis	Restricted to the assessment of SJS/TEN patients Liver damage is not taken into account in the scoring
**RegiSCAR** [37]	Assessment of hypersensitivity features and internal organ involvement Extensive use	Liver injury defined with low stringent thresholds
**CIOMS/RUCAM** [58]	High sensitivity, specificity, and predictive values Liver-specific	The presence of SCARs is not considered in the assessment High inter-rater variability Complex instructions
**Maria and Victorino** **[49]**	Easy-to-use Scores positively the presence of hypersensitivity features	No criterion related to internal organ involvement
**DDW-J** [48]	Based on the CIOMS/RUCAM Includes two new items related to hypersensitivity manifestations	Limited access Scores positively the performance of drug lymphocyte stimulation tests

ALDEN, Algorithm of Drug causality in Epidermal Necrolysis; WHO-UMC, World Health Organization—Uppsala Monitoring Centre; CIOMS/RUCAM, Council for International Organizations of Medical Sciences/Roussel Uclaf Causality Assessment Method; DDW-J, Digestive Disease Week-Japan; ADR, Adverse Drug Reaction; SJS: Steven–Johnson Syndrome; TEN, Toxic Epidermal Necrosis; DILI, Drug-Induced Liver Injury; SCARs, Severe Cutaneous Adverse Reactions.

## Data Availability

Not applicable.

## Supplementary Materials

The following are available online at https://www.mdpi.com/article/10.3390/jcm10225317/s1, Table S1: Revised Electronic Causality Assessment Method modified items.
