# Supplementary material for: Critical Review of Gaps in the Diagnosis and Management of Drug-Induced Liver Injury Associated with Severe Cutaneous Adverse Reactions
Source: J Clin Med. 2021 Nov 15;10(22):5317. doi: 10.3390/jcm10225317 (PMC8618381; doi:10.3390/jcm10225317)
Supplement: Supplementary file 1 [file jcm-10-05317-s001.zip › jcm-1456112-supplementary.pdf]

Supplemental Table S1. Revised Electronic Causality Assessment Method modified items.

Domains	RECAM modifications
Latency time	The domain was revised according to DILIN and Spanish DILI Registry data to optimize clarity, performance, and computerization
Dechallenge time	The domain was revised according to DILIN and Spanish DILI Registry data to optimize clarity, performance, and computerization
Risk factors	This domain was eliminated in the RECAM scale
Concomitant drugs	This domain was eliminated in the RECAM scale
Competing diagnoses	Competing diagnoses from RUCAM were retained and expanded
Drug specific risk	The scoring was anchored to LiverTox likelihood scores*
Rechallenge. Additional data	Rechallenge information was included in a new domain with additional optional information, such as histology or presence of DRESS or SJS

Hayashi et al. A revised electronic version of RUCAM for the diagnosis of drug induced liver injury. Hepatology 2020; 72(S1): 712A.

* LiverTox webpage: https://www.ncbi.nlm.nih.gov/books/NBK547852
